# Facilitating myoelectric-control with transcranial direct current stimulation: a preliminary study in healthy humans

**DOI:** 10.1186/1743-0003-11-13

**Published:** 2014-02-10

**Authors:** Anirban Dutta, Walter Paulus, Michael A Nitsche

**Affiliations:** 1 Department of Clinical Neurophysiology, Georg-August-University, Robert-Koch-Straße 40, Göttingen D-37075, Germany; 2 DEMAR team of INRIA, (Institut National de recherche en Informatique et Automatique) / LIRMM (Laboratoire d'Informatique, Robotique et Microélectronique de Montpellier), Montpellier 34090, France

**Keywords:** Functional electrical stimulation, Myoelectric control, Transcranial direct current stimulation, Stroke, Brain machine interfaces

## Abstract

**Background:**

Functional Electrical Stimulation (FES) can electrically activate paretic muscles to assist movement for post-stroke neurorehabilitation. Here, sensory-motor integration may be facilitated by triggering FES with residual electromyographic (EMG) activity. However, muscle activity following stroke often suffers from delays in initiation and termination which may be alleviated with an adjuvant treatment at the central nervous system (CNS) level with transcranial direct current stimulation (tDCS) thereby facilitating re-learning and retaining of normative muscle activation patterns.

**Methods:**

This study on 12 healthy volunteers was conducted to investigate the effects of anodal tDCS of the primary motor cortex (M1) and cerebellum on latencies during isometric contraction of tibialis anterior (TA) muscle for myoelectric visual pursuit with quick initiation/termination of muscle activation i.e. *'ballistic EMG control’* as well as modulation of EMG for *'proportional EMG control’*.

**Results:**

The normalized delay in initiation and termination of muscle activity during post-intervention *'ballistic EMG control’* trials showed a significant main effect of the anodal tDCS target: cerebellar, M1, sham (F(2) = 2.33, p < 0.1), and interaction effect between tDCS target and step-response type: initiation/termination of muscle activation (F(2) = 62.75, p < 0.001), but no significant effect for the step-response type (F(1) = 0.03, p = 0.87). The post-intervention population marginal means during *'ballistic EMG control’* showed two important findings at 95% confidence interval (critical values from Scheffe’s S procedure): 1. Offline cerebellar anodal tDCS increased the delay in initiation of TA contraction while M1 anodal tDCS decreased the same when compared to sham tDCS, 2. Offline M1 anodal tDCS increased the delay in termination of TA contraction when compared to cerebellar anodal tDCS or sham tDCS. Moreover, online cerebellar anodal tDCS decreased the learning rate during *'proportional EMG control’* when compared to M1 anodal and sham tDCS.

**Conclusions:**

The preliminary results from healthy subjects showed specific, and at least partially antagonistic effects, of M1 and cerebellar anodal tDCS on motor performance during myoelectric control. These results are encouraging, but further studies are necessary to better define how tDCS over particular regions of the cerebellum may facilitate learning of myoelectric control for brain machine interfaces.

## Background

Functional electrical stimulation (FES) can electrically activate a set of muscles selected to address individual movement deficits with a pre-programmed pattern of electrical stimulation [1,2]. Users normally employ a switch to manually trigger each pre-programmed stimulation pattern, but triggering and/or modulation of the electrical stimulation using residual electromyogram (EMG) from the paretic muscle - which is an alternative option to control FES - may encourage sensory-motor integration, where the residual volitional effort is reinforced with FES-assisted functional movement [3], and thus fostering re-learning of self-initiated movements. Unfortunately the muscle activity in hemiparetic limbs often suffers from a lack of coordination and delays in initiation/termination [4]. These deficits, which likely are controlled by the central nervous system (CNS), might be alleviated with an appropriate adjuvant treatment that improves CNS function. One possibly suited adjuvant treatment at the CNS level to facilitate learning of myoelectric control for brain machine interfaces is transcranial direct current stimulation (tDCS), which induces cortical excitability changes [5-8] induces neuroplasticity [9], and has been shown to improve motor learning in healthy humans [10,11], as well as in stroke survivors [12,13]. Therefore tDCS in combination with rehabilitative therapy has been suggested for stroke rehabilitation [14-16]. However tDCS-facilitated motor learning in lower limbs has not been explored systematically. Its effects on initiation and termination of muscle activations need further investigation to determine an appropriate adjuvant treatment with tDCS that may help in facilitating myoelectric control of FES. Tanaka and colleagues [17] found that anodal tDCS of the primary motor cortex representation of the tibialis anterior (TA) muscle (M1) had no significant effects on reaction time, but transiently enhanced maximal leg pinch force. Also, Madhavan and colleagues [18] found that M1 anodal tDCS of the primary motor representation of TA muscle applied to the lesioned motor cortex of moderate to well recovered stroke patients enhanced voluntary control of the paretic ankle. However, Galea and colleagues [19,20] did not observe any changes of reaction time with either M1 or cerebellar anodal tDCS.

**Figure 1:**
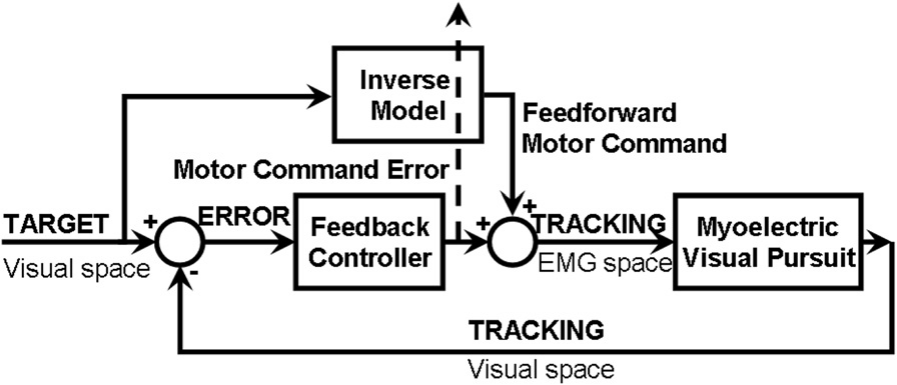
Schematic drawing of the cerebellar feedback-error-learning model: a feedback controller transforms sensory error in visual space into a feedback motor command which is used to train the inverse model for feedforward control.

In order to understand and further investigate the effects of tDCS on EMG latencies, we followed the general feedback-error-learning model [21] where both, the M1 and the cerebellum, are presumed to mediate generation of force profiles during manual tasks [22,23], as illustrated in Figure 1. The model incorporates three basic elements: 1. an inverse model that captures the feedforward part, 2. a feedback controller that captures the feedback part, and 3. a learning rule that adapts the inverse model based on motor command errors. The inverse (feedforward) model is primarily associated with the cerebellum and the feedback controller is primarily associated with premotor/motor cortices [23]. In this study, we investigated volitonal control of EMG during isometric conditions that reflects muscle force quite well [24]. Specifically, we investigated the impact of anodal tDCS of M1 and cerebellum on two commonly used myoelectric control paradigms for FES control [25], which are initiation/termination of muscle activation, i.e., *'ballistic EMG control*’ for switching FES on-off with a threshold-based classifier [26] and modulation of EMG for *'proportional EMG control’* of FES [27]. The myoelectric visual biofeedback was presented with proportional system dynamics, where the subjects had to modulate the EMG activity (here, EMG is the system input) from one level to another in a finite time. In this randomized sham-controlled study, we specifically explored two cases: 1. the effects of offline anodal tDCS of M1 and cerebellum on delays in initiation (step-up response) and termination (step-down response) of muscle activity to a visual on/off cue with maximal contraction of isometric TA muscle during *'ballistic EMG control’*, 2. the effects of online anodal tDCS of M1 and cerebellum on learning visual pursuit while following a sinusoidal target with EMG from TA during *'proportional EMG control’*.

## Methods

### Subjects

Twelve healthy right leg dominant male volunteers (age: 24–36 years) provided informed consent for this study. All the experiments were approved by the local ethics committee of the University Medicine Goettingen and conducted in accordance with the Declaration of Helsinki. Since most people display a dominance of kicking ability on one side, we assigned that side their dominant side. Determination of leg dominance is relevant, because it is thought to be associated with cortical movement representations, and might thus be a source of variability of lower limb motor function. Participants had no known neurological or psychiatric history, nor any contraindications to tDCS. One subject out of the total 12 subjects did not participate in Experiment 1 due to personal reasons.

### tDCS intervention

**Figure 2:**
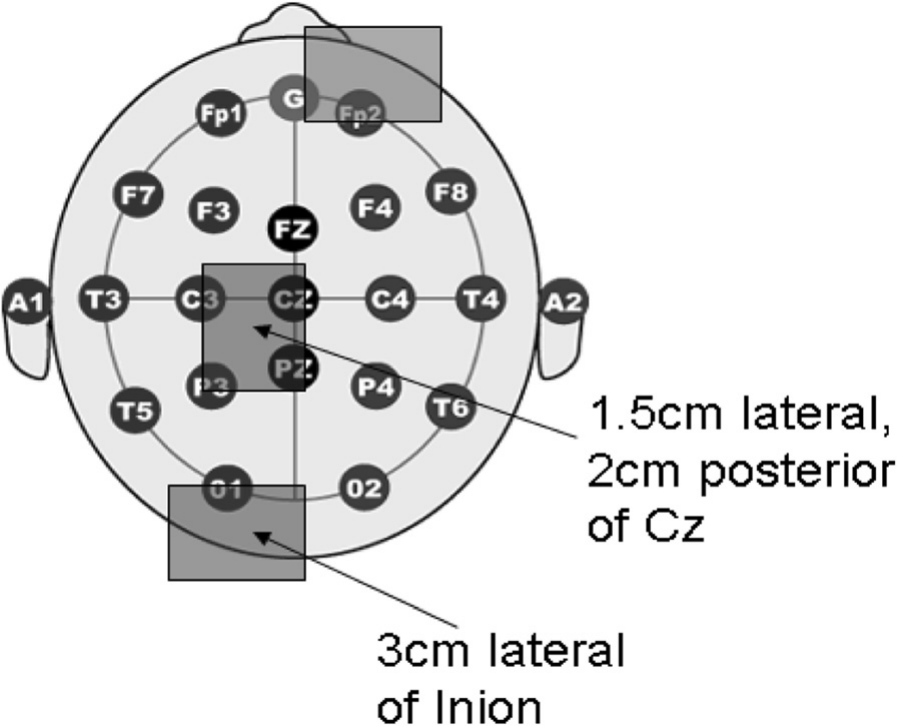
**Electrode montages for anodal tDCS (1 mA direct current for 15 min) of 1. primary motor cortex representation area of the right leg, where a 5 cm × 7 cm saline-soaked sponge anode was placed 1.5 cm lateral and 2 cm posterior to Cz (10–20 EEG system), 2. cerebellum of left hemisphere where the 5 cm × 7 cm saline-soaked sponge anode was placed 3 cm lateral to Inion (10–20 EEG system).** The 5 cm × 7 cm saline-soaked sponge cathode was placed above the right contralateral orbit.

Figure 2 shows the electrode montages for anodal tDCS (1 mA direct current for 15 min per session) via 2 saline-soaked 5 cm × 7 cm sponge electrodes with a DC-stimulator (NeuroConn, Germany). The stimulating anode was placed, 1) 1.5 cm left lateral and 2 cm posterior to Cz (10–20 EEG system) for targeting the primary motor cortex (M1) representation of the right leg TA muscle [28], 2) 3 cm left and lateral to the Inion (10–20 EEG system) for targeting the Cerebellar left hemisphere [19]. The cathodal return electrode was placed on the forehead above the right supraorbital ridge. During sham stimulation, the current was ramped up and then down to zero in 10 sec to provide blinding effects.

### Data collection and analysis

**Figure 3:**
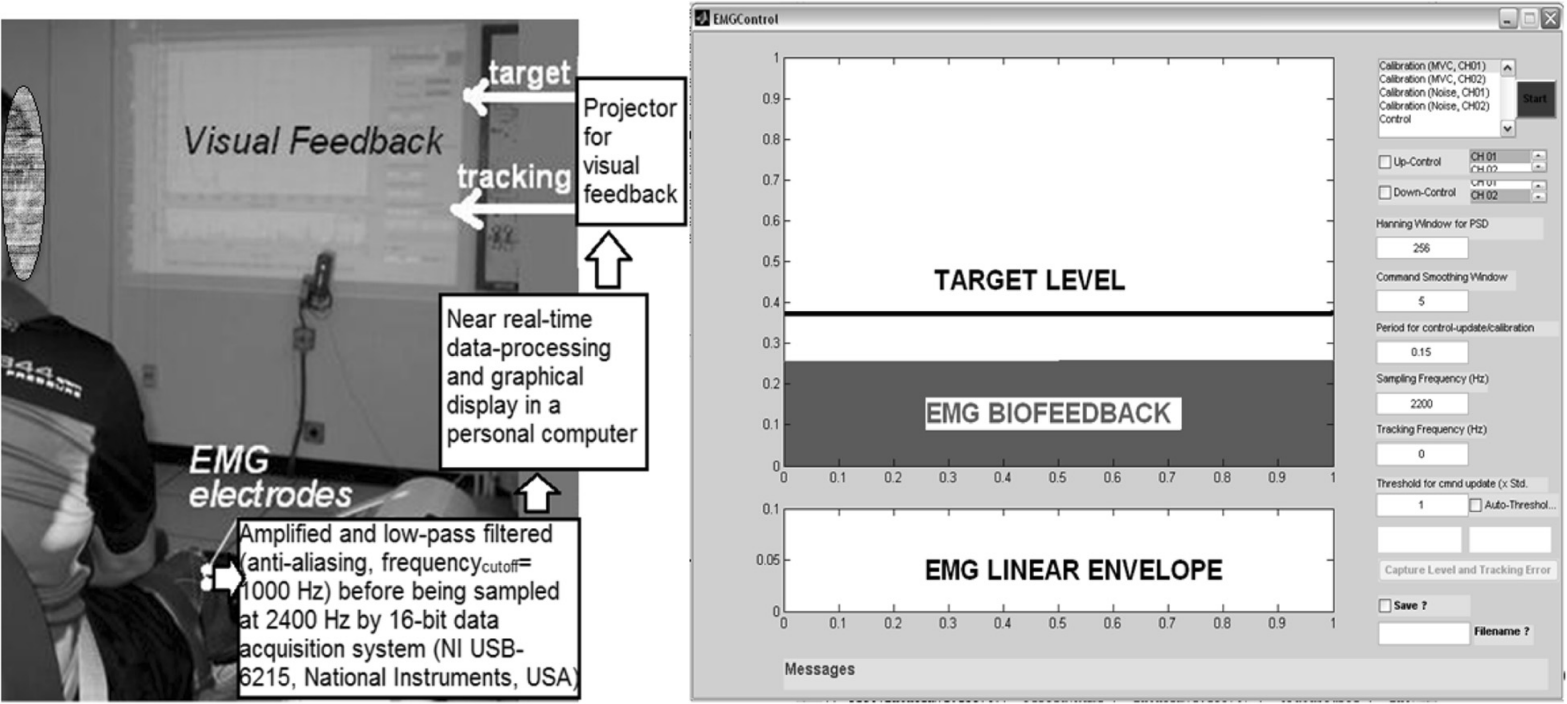
**Experimental setup for myoelectric control with visual feedback.** The normalized electromyogram (EMG) from tibialis anterior muscle was displayed as the TRACKING signal along with a sinusoidal TARGET signal.

The experimental setup for myoelectric control with visual feedback is shown in Figure 3. Surface EMG was collected from the TA muscle, amplified and low-pass filtered (anti-aliasing, frequency_cutoff_ = 1000 Hz) before being sampled at 2400 Hz by a 16-bit data acquisition system (NI USB-6215, National Instruments, USA) in a PC. Data-processing and graphical (GUI) display were performed with Matlab R2010a (The MathWorks, Inc., USA) using the Psychophysics Toolbox extensions [29-31]. The sampled EMG in a 400 ms moving window was digitally band-pass filtered (5th order zero-lag Butterworth, 20–500 Hz), de-trended, and rectified before being evaluated as a command signal (i.e., the TRACKING signal). A moving average of 400 ms of rectified EMG was found to provide appropriate smoothing for the EMG control task [32]. The average rectified EMG during one second of maximum voluntary isometric contraction (MVC) was used for normalization. Then, an estimate of the resting-state baseline EMG activity was set as one standard deviation over the average magnitude of the rectified EMG over one second while the subject was asked to relax the muscle. During visual pursuit tasks, the moving average of the rectified EMG was provided as visual feedback when it exceeded the resting-state baseline EMG activity. The normalized EMG was displayed as the TRACKING signal along with the TARGET signal (Figure 3). Both the TARGET signal that goes from 0 to 1 and the TRACKING signal (i.e., normalized EMG) pursuing the TARGET signal were updated at 100 Hz accounting for software-induced delays in processing, and were projected on the wall in front of the subject at the eye level, as illustrated in Figure 3.

**Figure 4:**
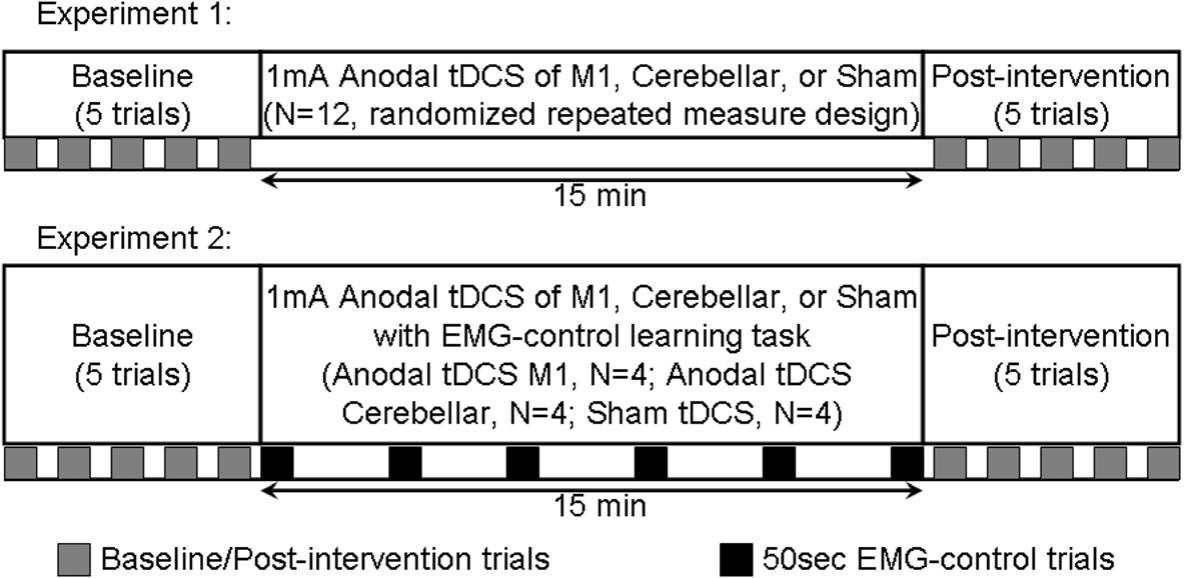
Experimental protocols for Experiment 1 (top panel): offline anodal tDCS, and Experiment 2 (bottom panel): online anodal tDCS with myoelectric visual pursuit, each with three one-day test sessions separated by at least a week, where subjects received 15 min of anodal tDCS to M1, cerebellum, or sham tDCS.

In the first one-day session, the subjects learned to isometrically contract the TA muscle as quickly and as forcefully as possible, in response to a visual cue when the TARGET signal jumped from 0 to 1 (step-up response), while the ankle was kept fixed in an ankle-foot-orthosis (AFO). Then the subjects learned to relax the TA muscle as quickly as possible on termination of the visual cue when the TARGET signal jumped from 1 to 0 (step-down response). After the subjects were comfortable with this step-up and then step-down evaluation procedure, they participated in two sets of experiments, as illustrated in Figure 4, with each one-day test session separated by at least a week.

#### 1) Experiment 1

11 subjects performed five baseline trials (i.e., baseline task block) where they responded with quick and forceful contraction of the TA muscle to a visual cue when the TARGET signal jumped from 0 to 1 (step-up response), and then quickly relaxed the TA muscle on termination of the visual cue when the TARGET signal jumped from 1 to 0 (step-down response). The visual cue duration was either 3 sec, 4 sec, or 5 sec, during which the subject had to maintain the TRACKING signal as close as possible to the TARGET signal. The visual cues were presented in a pseudorandom order with a random 3 sec, 4 sec, or 5 sec inter-cue-interval. Following the baseline task block, 15 min of 1 mA anodal tDCS was administered to M1, cerebellum, or under sham stimulation in a repeated measure counter-balanced design, after which the subjects performed 5 post-intervention trials (i.e., post-intervention task block) similar to the baseline trials.

During offline analysis in Matlab R2010a (The Mathworks Inc., USA), the raw EMG sampled during each task block of the experiment was digitally zero-phase band-pass filtered (5th order Butterworth, 3 dB bandwidth = 10-500 Hz), then full-wave rectified, and then zero-phase low-pass filtered (5th order Butterworth, 3 dB frequency_cutoff_ = 25 Hz) to generate its linear EMG-envelope (LE). The delay in initiation of the EMG LE was defined manually as the time interval between onset of the visual cue and the instant the LE crossed above baseline LE (i.e., mean resting-state LE + 1 standard deviation). The delay in termination of the EMG LE was defined manually as the time interval between termination of the visual cue and the instant the LE crossed below baseline LE. Each LE tracing was displayed on a PC monitor in random order without reference to subject, cue duration, or tDCS targets, in order to reduce relative bias.

#### 2) Experiment 2

12 subjects performed five baseline trials (i.e., baseline task block) similar to the baseline task block of Experiment 1. After the baseline trials, the subjects were randomly divided into M1, cerebellum, or sham stimulation groups. Then they performed the myoelectric visual pursuit task for 15 min. 1 mA anodal tDCS was simultaneously administered to M1, cerebellum, or sham stimulation was performed (i.e. 4 subjects per group). The subjects were asked to track the absolute value of a sinusoid of 0.7 amplitude and 0.01 Hz frequency over its half time-period (i.e., the TARGET signal) during each trial. A set of six of such consecutive 50 sec trials with 2 min of rest in-between, were presented to the subjects during the administration of anodal/sham tDCS.

During offline analysis in Matlab R2010a (The Mathworks Inc., USA), the response latency was computed from the delay in initiation of the TRACKING signal with respect to the start of the TARGET signal where the initiation was defined as the instant the TRACKING signal crossed baseline EMG activity. Then, the response latency was normalized by subjects’ respective mean baseline delay over their 5 baseline trials. Each TRACKING signal tracing was displayed on a PC monitor in random order without reference to subject or tDCS targets, in order to reduce relative bias. The absolute value of the difference between the TARGET and TRACKING signals, i.e., the tracking-error signal (ERROR signal = |TARGET signal ‒ TRACKING signal|) was computed after removing the response latency from the TRACKING signal, where the mean of the absolute ERROR was analyzed as a measure of tracking accuracy.

### Statistical analysis

#### 1) Experiment 1

The delays in initiation and termination of muscle activity during baseline and post-intervention trials were tested for normal distribution by the univariate Lilliefors test ('lillietest’ in Matlab R2010a, The MathWorks, Inc., USA) for sessions of each tDCS target - M1, cerebellar, sham - pooled from all subjects. Then, a balanced three-way (tDCS target: M1, cerebellar, sham x step-response type: step-up, step-down x subjects) ANOVA ('anovan’ in Matlab R2010a, The MathWorks, Inc., USA) was conducted on the step-response, i.e., the delay in initiation and termination of muscle activity during the baseline trials. Also, a balanced two-way (tDCS target: M1, cerebellar, sham × step-response type: step-up, step-down) ANOVA ('anova2’ in Matlab R2010a, The MathWorks, Inc., USA) was conducted on the normalized delay in initiation and termination of muscle activity during the post-intervention trials. The delay was normalized by subjects’ respective mean baseline delay over their 5 baseline trials. To find which pairs were significantly different, post hoc tests ('multcompare’ in Matlab R2010a, The MathWorks, Inc., USA) were performed with the critical values found from Scheffe’s S procedure.

#### 2) Experiment 2

The delays in initiation and termination of muscle activity during baseline trials were tested for normal distribution by the univariate Lilliefors test ('lillietest’ in Matlab R2010a, The MathWorks, Inc., USA) for each tDCS group - M1, cerebellar, sham. Then, a balanced two-way (tDCS target: M1, cerebellar, sham x step-response type: step-up, step-down) ANOVA ('anova2’ in Matlab R2010a, The MathWorks, Inc., USA) was conducted on the step-response, i.e., the delay in initiation and termination of muscle activity during the baseline trials.

The normalized response latency and the mean absolute ERROR during the last 5 myoelectric visual pursuit trials (Trial# 2–6) were assessed by fitting the performance with a power law function [33] using the Levenberg-Marquardt algorithm ('cftool’ in Matlab R2010a, The MathWorks, Inc., USA). The 95% confidence bounds of the coefficients of the fitted power law function were compared for the tDCS groups: M1, cerebellar, sham.

## Results

### 1) Experiment 1

The delays in initiation and termination of muscle activity during baseline trials passed the Lilliefors test for normal distribution at 5% significance level for pooled sessions of each tDCS target - M1, cerebellar, sham. The balanced three-way (tDCS target: M1, cerebellar, sham × step-response type: step-up, step-down × subjects) ANOVA on the delay in initiation and termination of muscle activity during baseline trials showed a significant main effect of the step-response type (F(1) = 2597.11, p < 0.001), but no significant effect for other factors, tDCS target (F(2) = 0.55, p = 0.58), subjects (F(10) = 0.87, p = 0.56), or the interaction effect between the tDCS target and the step-response type (F(2) = 0.01, p = 0.99), the interaction effect between tDCS target and subjects (F(20) = 1.35, p = 0.15), the interaction effect between step-response type and subjects (F(10) = 1.1, p = 0.36). The post hoc tests performed with the critical values found from Scheffe’s S procedure confirmed that for the step-response type, the termination of muscle activity was significantly slower (95% confidence interval for mean delay: 590 ms to 603 ms) than initiation of the muscle activity (95% confidence interval for mean delay: 241 ms to 255 ms) during the baseline trials.

**Figure 5:**
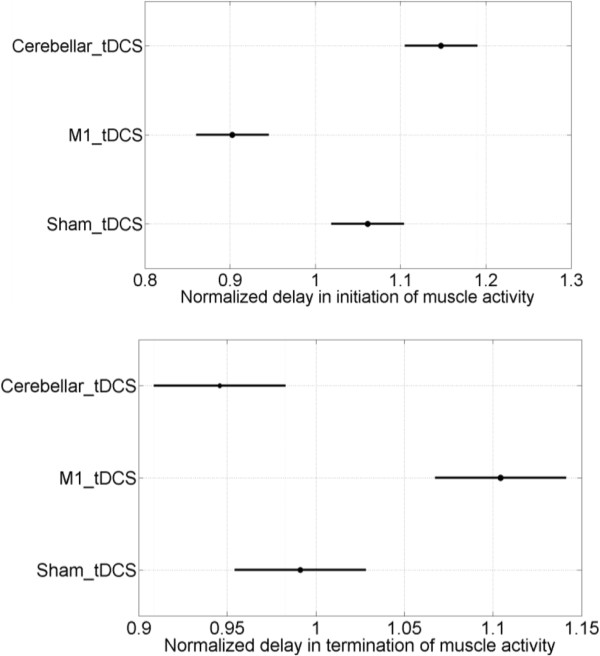
**The post hoc comparisons in the normalized delay in initiation (top panel) and termination (bottom panel) of muscle activity to the grouping variable, tDCS target: M1, cerebellum, sham, during *'ballistic EMG control’ *for Experiment 1.** The differences were considered significant if 95% confidence intervals represented by a line for the mean shown by a filled circle did not overlap.

The delays in initiation and termination of muscle activity during post-intervention trials passed the Lilliefors test for normal distribution at 5% significance level for pooled sessions of each tDCS target - M1, cerebellar, sham. The balanced two-way (tDCS target: M1, cerebellar, sham x step-response type: step-up, step-down) ANOVA on the normalized delay in initiation and termination of muscle activity during post-intervention trials showed a significant main effect of the tDCS target (F(2) = 2.33, p < 0.1), and interaction effect between tDCS target and step-response type (F(2) = 62.75, p < 0.001), but no significant effect for the step-response type (F(1) = 0.03, p = 0.87). With the critical values found from Scheffe’s S procedure, the 95% confidence interval for the mean normalized delay in initiation and termination of muscle activity during post-intervention trials are shown separately in Figure 5. The differences in the normalized delay in initiation of muscle activity were significantly different for all factor levels of tDCS target, with cerebellar anodal tDCS increasing and M1 anodal tDCS decreasing it when compared to sham tDCS, but the differences in normalized delay in termination of the muscle activity were significantly different only for M1 anodal tDCS, which increased the normalized delay when compared to cerebellar anodal tDCS and sham tDCS. Cerebellar anodal tDCS trended towards decreasing the normalized delay in termination of the muscle activity when compared to sham tDCS.

### 2) Experiment 2

The delays in initiation and termination of muscle activity during baseline trials passed the Lilliefors test for normal distribution at 10% significance level for each tDCS group - M1, cerebellar, sham. The balanced two-way (tDCS target: M1, cerebellar, sham × step-response type: step-up, step-down) ANOVA on the delay in initiation and termination of muscle activity during baseline trials showed a significant main effect of the step-response type (F(1) = 506.89, p < 0.001), but no significant effect for the other factor, tDCS target (F(2) = 0.6, p = 0.55). The post hoc tests performed with the critical values found from Scheffe’s S procedure confirmed that for the step-response type, the termination of muscle activity was significantly slower (95% confidence interval for mean delay: 492 ms to 514 ms) than initiation of the muscle activity (95% confidence interval for mean delay: 230 ms to 253 ms) during the baseline trials.

**Figure 6:**
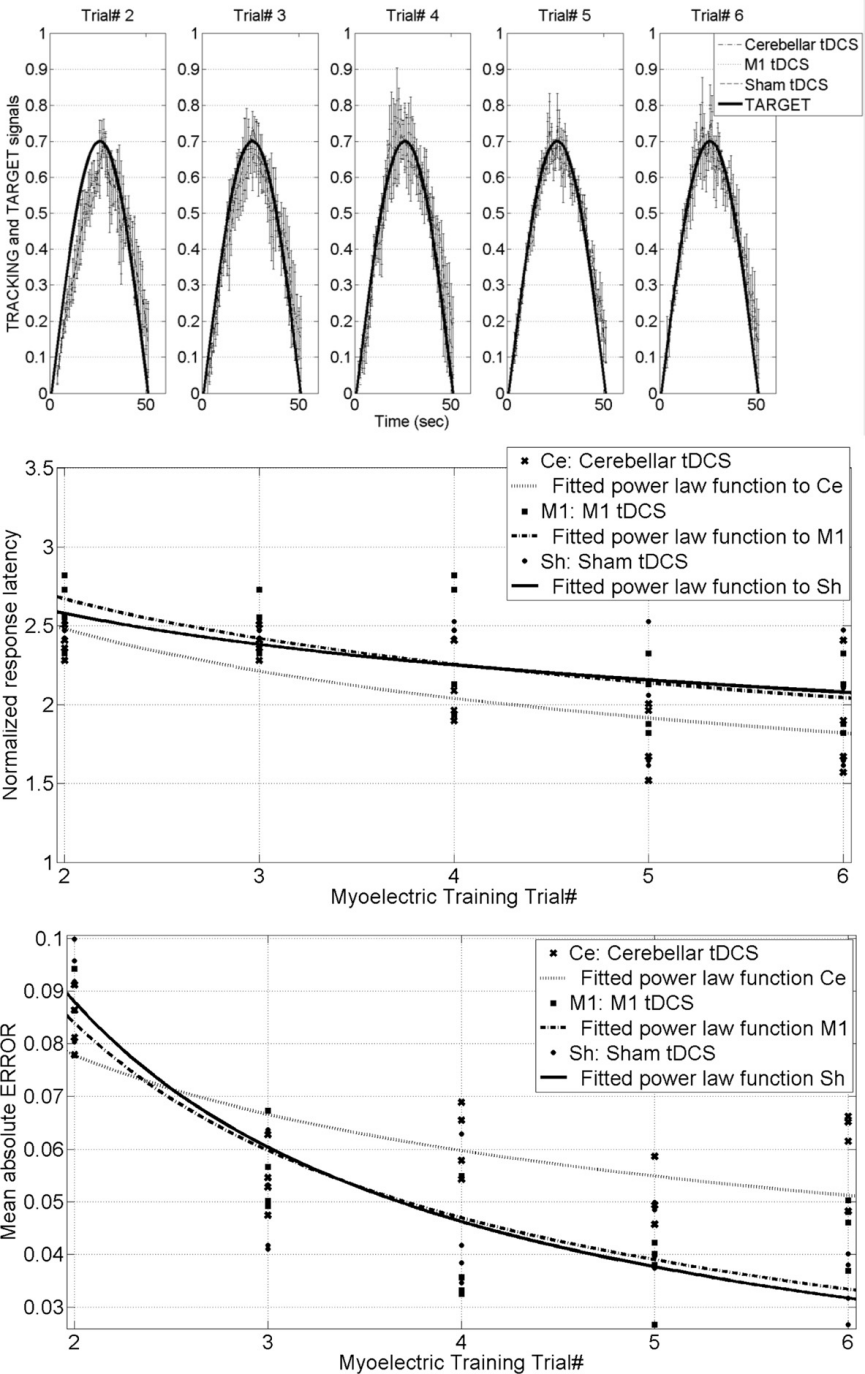
**Results from myoelectric the visual pursuit task (i.e., Experiment 2) for Myoelectric Training Trial# 2–6 for the tDCS groups: M1, cerebellum, sham.** The top row illustrates the overall TARGET and TRACKING signals during the modulation of EMG during *'proportional EMG control’* trials, the middle row shows the effects on the normalized response latency, and the bottom row shows the effects on the mean absolute ERROR.

The normalized response latency and the mean absolute ERROR during the last 5 myoelectric visual pursuit trials (i.e., Trial# 2–6) were assessed by fitting the performance with a power law function [33]. Figure 6 shows the results of the myoelectric visual pursuit task for the tDCS groups: M1, cerebellum, sham, and training-durations: Trial# 2–6. The top row of Figure 6 shows the overall TARGET and the TRACKING signals, the middle row shows the effects on the normalized response latency along with the fitted power law function, and the bottom row shows the effects on the mean absolute ERROR along with the fitted power law function. The 95% confidence bounds of the coefficients of the power law function fitted to normalized response latency and the mean absolute ERROR are provided in Table 1 for each tDCS group: M1, cerebellar, sham. Here the power law exponent for the mean absolute ERROR of the cerebellar tDCS group did not overlap with those of the other tDCS groups.

**Table 1:** The 95% confidence bounds of the coefficients of the power law function fitted to normalized response latency and the mean absolute ERROR for each tDCS group: M1, cerebellar, sham


**Response latency**	**a (95% confidence bounds)**	**b (95% confidence bounds)**	**SSE**	**R-square**	**RMSE**
Cerebellar tDCS	3.011 (2.476,3.546)	-0.2806 (-0.4182,-0.143)	1.091	0.4579	0.2462
M1 tDCS	3.158 (2.542,3.775)	-0.2426 (-0.3924,-0.09276)	1.557	0.3107	0.2941
Sham tDCS	2.951 (2.287,3.614)	-0.1952 (-0.366,-0.0243)	1.977	0.148	0.3314
**Mean absolute error**	**a (95% confidence bounds)**	**b (95% confidence bounds)**	**SSE**	**R-square**	**RMSE**
Cerebellar tDCS	0.1013 (0.07503,0.1275)	-0.3808 (-0.5862,-0.1754)	0.002164	0.3767	0.01097
M1 tDCS	0.1498 (0.1086,0.191)	-0.8368 (-1.082,-0.5918)	0.002198	0.7172	0.01105
Sham tDCS	0.1671 (0.1212,0.2129)	-0.9265 (-1.177,-0.676)	0.002279	0.758	0.01125

Power law function: f(x) = a*x^b^; SSE: sum of squares due to error; R-square: coefficient of determination; RMSE: root mean squared error (standard error).

## Discussion

In this study, the motor control task involved visual pursuit of a TARGET signal with EMG-based proportional control of a visual TRACKING signal. Prior work has shown that EMG reflects muscle force quite well during isometric conditions where EMG follows a quadratic increase in its root-mean-square value across force levels [24]. In Experiment 1, the myoelectric step-response task was familiar to the subjects but the trials were presented in an unpredictable temporal manner during baseline and post-intervention to avoid cognitive anticipation, and to identify the delay in initiation and termination of muscle activity during an open-loop *'ballistic EMG control’* task. We first ruled out subject specific effects on the initiation and termination of muscle activity during baseline trials, and then found that termination of muscle activity was significantly (p < 0.05) slower than initiation of muscle activity for all subjects over all baseline trials. We also found that cerebellar anodal tDCS increased the normalized delay in initiation of muscle activity post-intervention while M1 anodal tDCS decreased it, when compared to sham tDCS, as shown in the top panel of Figure 5. Also, M1 anodal tDCS increased the normalized delay in termination of muscle activity post-intervention when compared to cerebellar anodal tDCS and sham tDCS, as shown in the bottom panel of Figure 5. Therefore in this study, off-line anodal tDCS of M1 decreased the delay in initiation while it increased the delay in termination during performance of the *'ballistic EMG control’* task. However Galea and colleagues [20] did not observe any changes in reaction time with either M1 or cerebellar anodal tDCS during a more complex task where the subjects had to move a digitizing pen with their right hand over a horizontal digitizing tablet. The outcome differences could be caused by different EMG recordings in the respective studies. In the present study, EMG latencies were obtained only for the excitation dynamics of the target muscle. Excitation and contraction processes of multiple muscles crossing a joint and subsequent joint mechanics, as recorded in the study by Galea and colleagues [20], might limit comparability. Moreover, the premotor cortex might have played an important role in the rather complex (fine) motor task movements employed by Galea and colleagues [20] that required controlled contraction of multiple muscles compared to the control of a single muscle in this study. Here, M1 is involved in task performance in part through its reciprocal interaction with the cerebellum [34]. Cerebellar priming of M1 plasticity in shaping the impending motor command by favoring or inhibiting the recruitment of several muscle representations has been shown recently [35]. Prior work has suggested that cerebellar anodal tDCS may increase the Purkinje cells’ excitability and facilitate the inhibitory tone the cerebellum exerts over M1 (cerebellar brain inhibition) [19], which explains an increase in normalized delay in initiation of muscle activity following cerebellar anodal tDCS and a respective decrease following M1 anodal tDCS. Conversely, cerebellar-caused M1 inhibition should then facilitate sudden termination of ongoing muscle activity. In principal accordance with this concept, cerebellar anodal tDCS trended towards decreasing the normalized delay in termination of muscle activity post-intervention, while M1 anodal tDCS significantly increased it, when compared to sham tDCS.

In Experiment 2, naive subject learned a novel visual pursuit task. Subjects had to minimize spatial ERROR between the TARGET signal and the TRACKING signal using visual feedback. The response latency was high at the start of myoelectric training during *'proportional EMG control’*, but reduced with training as the TRACKING response signal temporally shifted with respect to the TARGET signal, as shown in the top panel of Figure 6. The visual pursuit was initially driven primarily with feedback motor command where the feedback motor command also served as the motor command error for developing or modifying the inverse model for this novel visuomotor transformation [23] as the subject learned proportional dynamics of the myoelectric visual pursuit. The feedback control is inherently slow because it uses delayed sensory (e.g., proprioceptive and visual) signals to compute the motor command [36,37] but feedforward control uses the inverse model to predict the (feedforward) motor command necessary to pursue the TARGET signal, which should increase the speed and accuracy during visual pursuit if the inverse model is accurate [21,36]. However, it was found that the absolute value of the power law exponent for the mean absolute ERROR of the cerebellar tDCS group was lower than other tDCS groups (see Table 1), which indicated slower motor learning (bottom panel of Figure 6) during *'proportional EMG control.’* This is in contrast with the results from Galea and colleagues [18], which may be explained in terms of a computational model of human motor learning. In the computational model of the cerebellar circuit, the simple spikes represent the feedforward motor commands and the parallel fiber inputs represent the desired trajectory (TARGET) as well as the sensory state of the TRACKING signal [21]. The climbing fiber inputs are assumed to carry a copy of the feedback motor commands where the complex spiking of the climbing fibers in the cerebellum are considered to be the biological representation of an error signal. Marko and colleagues [38] recently found that the probabilities of complex spiking declined with increasing error size and therefore they postulated that complex spiking is a representative of the sensitivity to error, and not the error itself. Therefore learning of the inverse model may be dependent on the sensitivity to error, in addition to the magnitude of the error presented during the task performance. From results of Experiment 1, it can be postulated that there was facilitation of cerebello-thalamocortical inhibitory connections at movement initiation [39] with cerebellar anodal tDCS, which might have reduced the sensitivity of the Purkinje cells to errors represented by complex spiking of the climbing fibers that resulted in a slower decrease in the mean absolute ERROR during *'proportional EMG control’* trials. In fact, Galea and colleagues [20] hypothesized that cerebellar tDCS may change Purkinje cells response to the input of the climbing fibers by affecting secondary events such as long-term depression. Moreover, Popa and colleagues recently showed that modulation of the cerebellar cortex by noninvasive brain stimulation affects the response of M1 to a subsequent plasticity induction protocol that involves sensory afferent input but not otherwise [35].

In terms of clinical applications, the current study on healthy subjects showed a decrease in the normalized delay in initiation of muscle activity following M1 anodal tDCS which may be beneficial for stroke survivors who often suffer from delays in initiation of muscle activity [4]. Therefore an adjuvant treatment with M1 anodal tDCS may facilitate appropriate myoelectric triggering of FES where delays in initiation of muscle activity makes it difficult for EMG-triggered FES to assist time-critical functional tasks such as ankle dorsiflexion during overground walking. However, the optimal positioning of the cerebellar tDCS electrode remains unclear. More studies are required to better define how tDCS over particular regions of the cerebellum affects individual cerebellar sensory-motor functions given its topographical organization [40]. In our future studies, we will investigate optimization of the electrode montage for cerebellar tDCS to target different sensory modalities (e.g., proprioceptive instead of visual), since recent studies in patients with cerebellar damage demonstrated that adaptation to proprioceptive versus visual errors relies on the integrity of different regions of the cerebellum [41,42]. Therefore anodal tDCS-induced changes in excitability of different regions of the cerebellum may differentially affect proprioceptive versus visual sensory modalities [40].

## Conclusions

The preliminary results from healthy subjects showed specific, and at least partially antagonistic effects, of M1 and cerebellar stimulation on motor performance. An appropriate adjuvant treatment with tDCS may help to facilitate myoelectric control for brain machine interfaces, however the neuroprosthetic and neurotherapeutic efficacy of such an adjuvant treatment needs further investigation in stroke survivors. Furthermore, an adjuvant treatment with tDCS may improve muscle recruitment and coordination during post-stroke neurorehabilitation.

## Competing interests

A. Dutta and W. Paulus declare that they have no competing interests. M.A. Nitsche declares that he is in the Advisory Board of Neuroelectronics, S.L., Spain.

## Authors’ contributions

AD have made substantial contributions to conception and design of the experiments, acquisition, analysis and interpretation of data. AD have been involved in drafting the manuscript. WP and MAN have made contributions to conception and design of the experiments, in revising the manuscript critically for important intellectual content; and have given final approval of the version to be published. All authors read and approved the final manuscript.
